# Novel method for modified interlaminar approach using contralateral oblique view: A technical suggestion

**DOI:** 10.1371/journal.pone.0244992

**Published:** 2021-01-06

**Authors:** Jungwon Baek, Jia Kim, Seunghee Cho, Yujin Jeong, Eung Don Kim

**Affiliations:** 1 Department of Anesthesiology and Pain Medicine, Incheon St. Mary’s Hospital, College of Medicine, The Catholic University of Korea, Seoul, Republic of Korea; 2 Department of Anesthesiology and Pain Medicine, Daejeon Veterans Hospital, Daejeon, Republic of Korea; 3 Department of Anesthesiology and Pain Medicine, Daejeon St. Mary’s Hospital, College of Medicine, The Catholic University of Korea, Seoul, Republic of Korea; Heidelberg University Hospital, GERMANY

## Abstract

A modified interlaminar (MIL) approach has been proposed for improved accessibility to the target epidural space. However, even with fluoroscopic guidance, uncertainty about the distance between the needle tip and the epidural space can remain. Using the contralateral oblique (CLO) view, determination of the epidural space can be easier with clearer identification of the interlaminar opening. We inserted the needle at the midpoint of the interlaminar opening on the fluoroscopic anteroposterior (AP) view and made the needle oriented toward the pedicle of the target side. Then, CLO view was created by rotating the intensifier approximately 45 degrees to the contralateral side of the target. Through the CLO view, the ventral interlaminar line (VILL) was confirmed and the needle was able to enter the epidural space more easily. The medical records of 29 patients who were conducted MIL approach using CLO view were retrospectively analyzed to evaluate the effectiveness and safety of this procedure. The accessibility to the ventral epidural space was 93.1%. There was no procedure-related complication. Using CLO view, uncertainty can be reduced during the MIL approach, which in turn shortens procedure time and improves safety.

## 1. Introduction

Interlaminar (IL) epidural block is widely used for treatment of spinal pain [1]. In many cases, the cause of spinal pain is located in the ventral epidural space [2]. Since accessibility to the ventral epidural space through the conventional IL approach may be restricted [3], a transforaminal (TF) approach has been proposed and widely used [4]. However, it may be difficult to access the target lesion through the TF approach in cases of degenerative changes of the spine such as an enlarged articular process.

**Fig 1:**
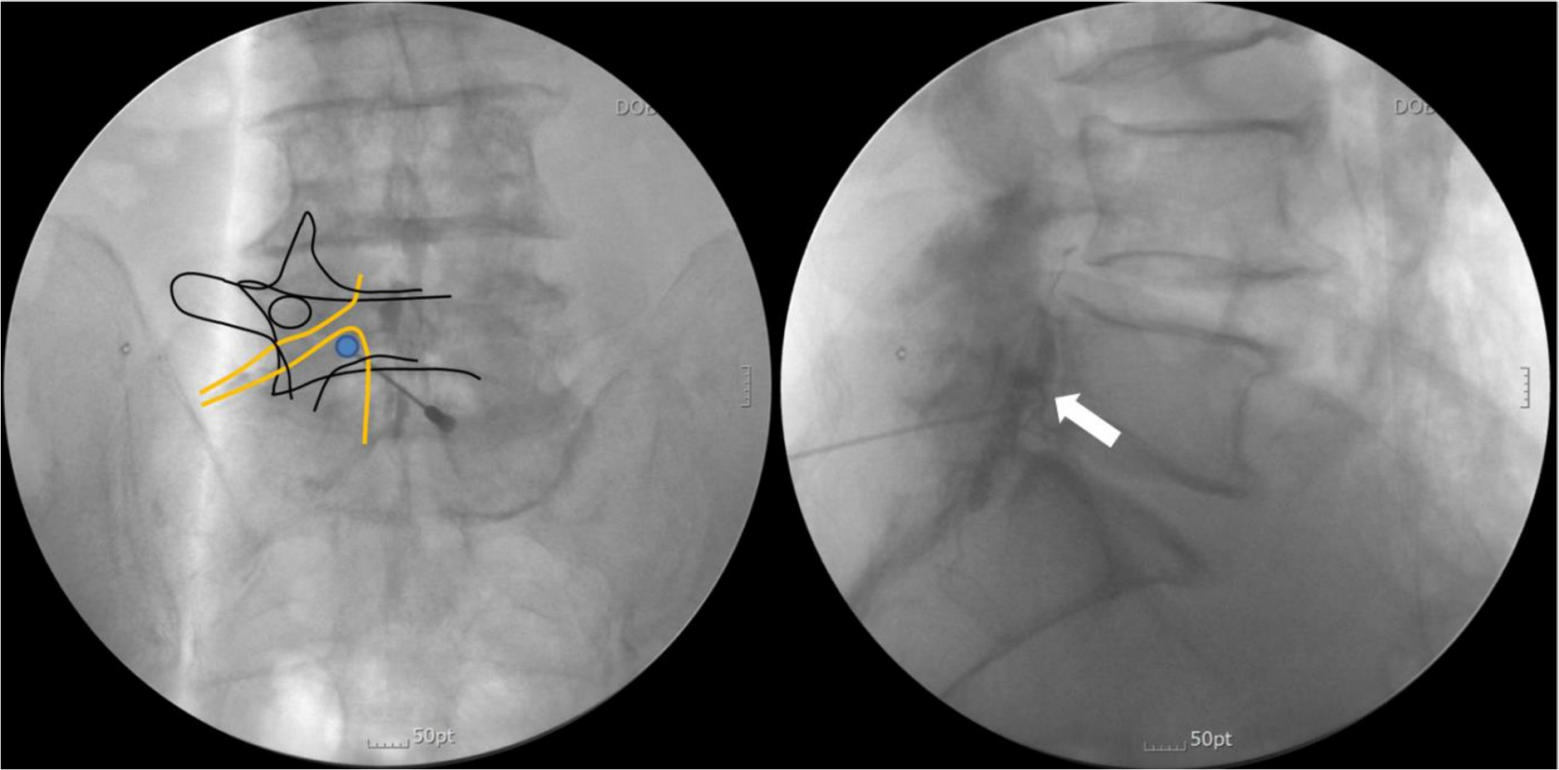
The final needle tip position in the modified interlaminar (MIL) approach is the ventral epidural space of the axillary portion of the exiting nerve root. The filled circle indicates the position of the needle tip, while the white arrow indicates the ventral spread of the contrast medium.

For better accessibility to the target epidural space, we proposed a modified interlaminar (MIL) approach [5]. The final goal of the MIL approach is to place the needle tip in the ventral epidural space of the axillary portion of the exiting nerve root (Fig 1). This technique requires the needle to be advanced toward the pedicle and touch the superolateral margin of the interlaminar opening in fluoroscopic anteroposterior (AP) view before entering the epidural space [5]. However, it is difficult to estimate the depth of the needle tip in fluoroscopic AP view; even in fluoroscopic lateral view, it is often difficult to predict the exact location of the epidural space.

Contralateral oblique (CLO) view is a proposed method to facilitate the IL approach at the cervicothoracic level. Through CLO view, the ventral interlaminar line (VILL) can be easily detected and, by more clearly identifying the lamina, determination of epidural space can be easier [6].

We would like to propose a method to perform the MIL approach more easily and safely using CLO view in the lumbar spine. For this purpose, we retrospectively analyzed the medical records of patients who underwent the MIL approach using CLO view.

## 2. Methods

Permission to conduct this study was obtained from the Institutional Ethics Committee of Daejeon St. Mary’s Hospital, Republic of Korea (DC20RISI0064). The medical records of patients who were conducted MIL approach using CLO view between January 2019 and June 2020 were collected.

### 2.1. Patient selection

We usually performed epidural block in patients who complained of numerical rating scale (NRS) 3 or more lumbar radicular pain over 18 years of age. When spreading of injectate to ventral epidural space or medial epidural space was restricted through TF approach, MIL approach was attempted.

### 2.2. Procedure protocol

**Fig 2:**
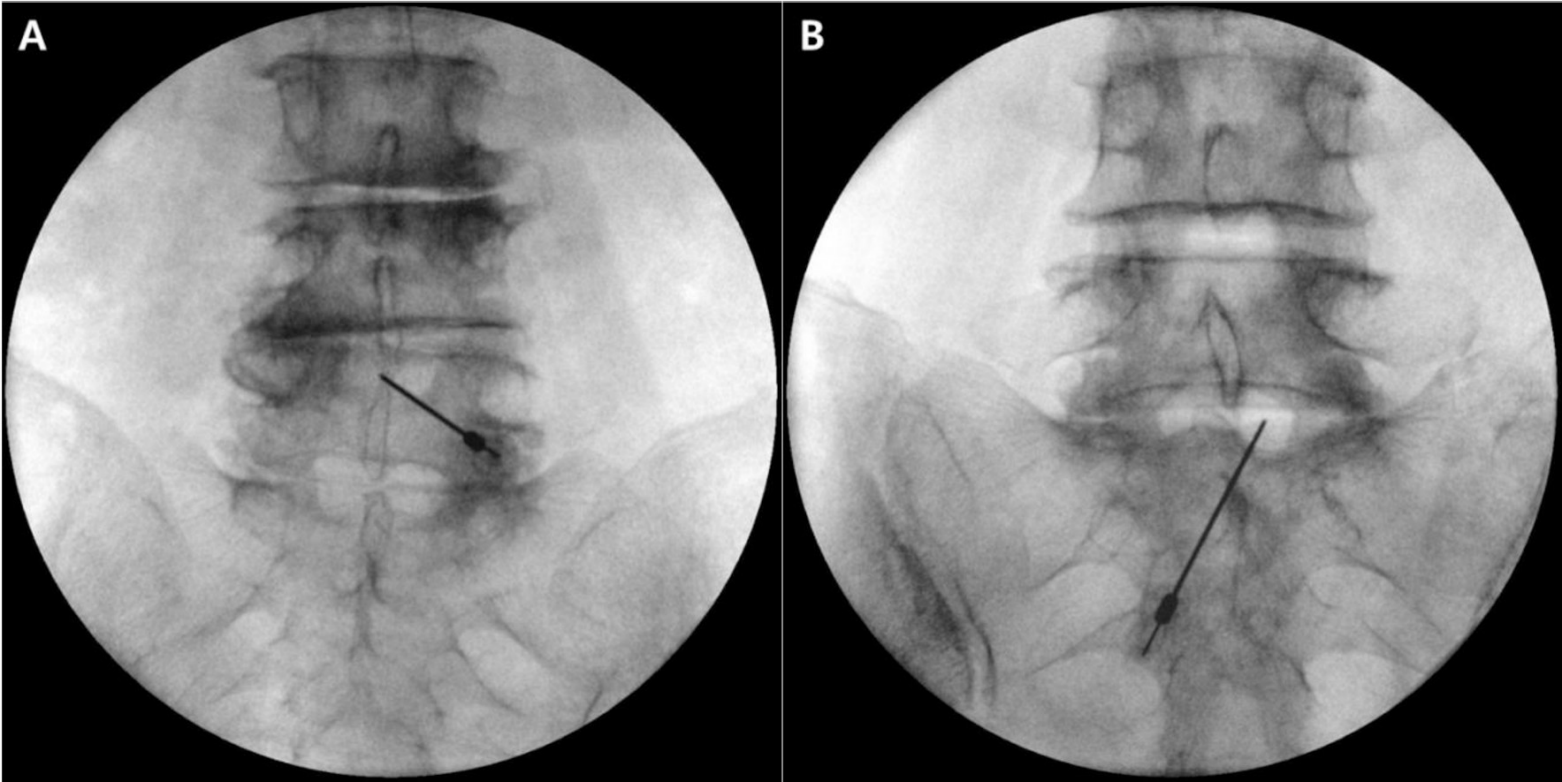
Advancement of the needle toward the pedicle of the target side. The target point of A is the axillary portion of the left L4 root, and that of B is the axillary portion of the right L5 root.

After providing written informed consent, the patient was placed in the prone position, and the needle entry site was created at the midpoint of the lower margin of the interlaminar opening or slightly below it in fluoroscopic AP view. After local anesthetic infiltration, a 20-gauge Tuohy needle was advanced and oriented toward the pedicle of the target side (Fig 2).

**Fig 3:**
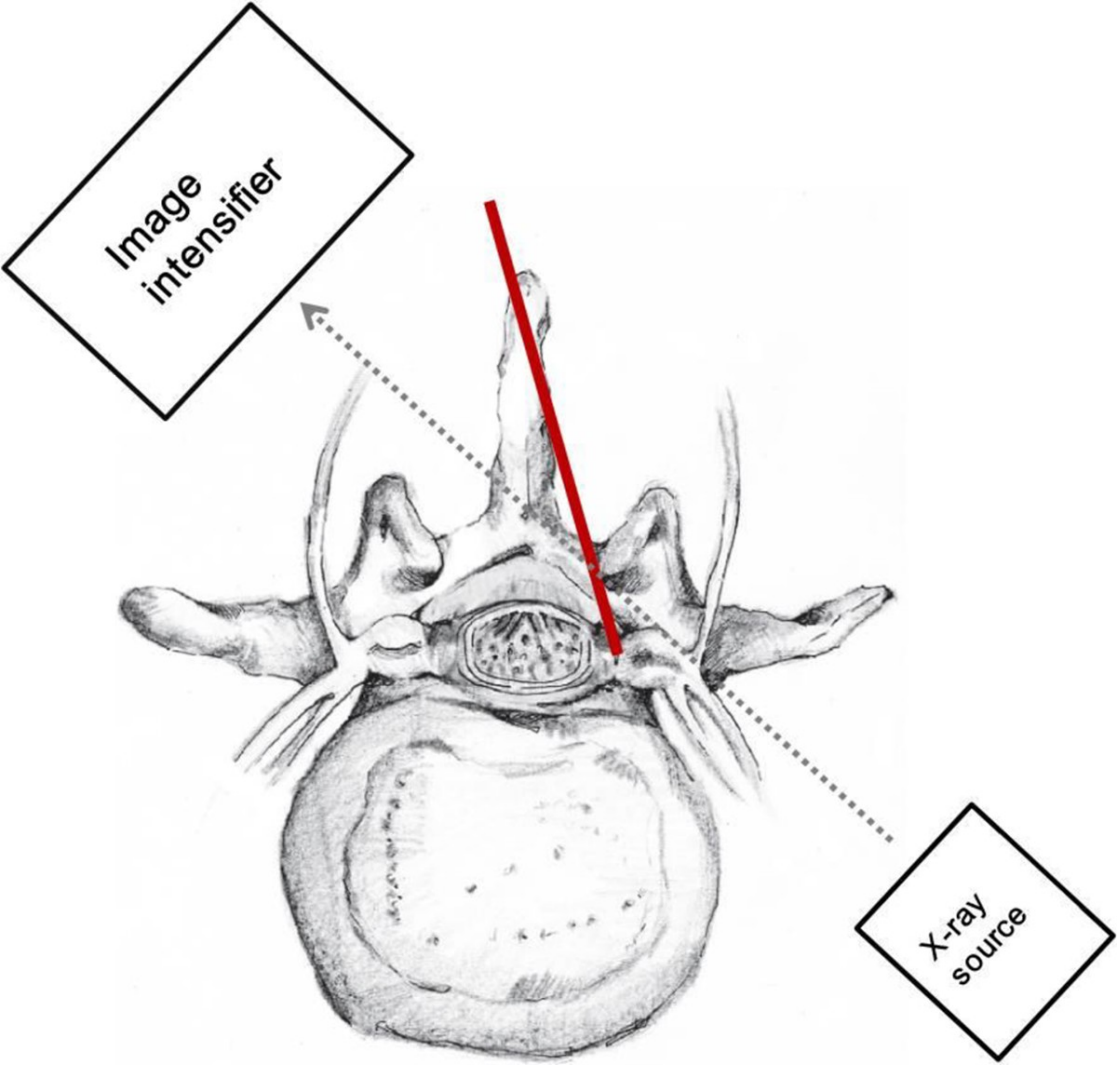
Demonstration of fluoroscopy location to create contralateral oblique (CLO) view during the modified interlaminar (MIL) approach. The intensifier was rotated approximately 45 degrees contralateral to the target side. The red line indicates the needle direction. The dotted arrow indicates the direction of the X-ray beam.

CLO view was created by rotating the intensifier approximately 45 degrees to the contralateral side of the target (Fig 3).

**Fig 4:**
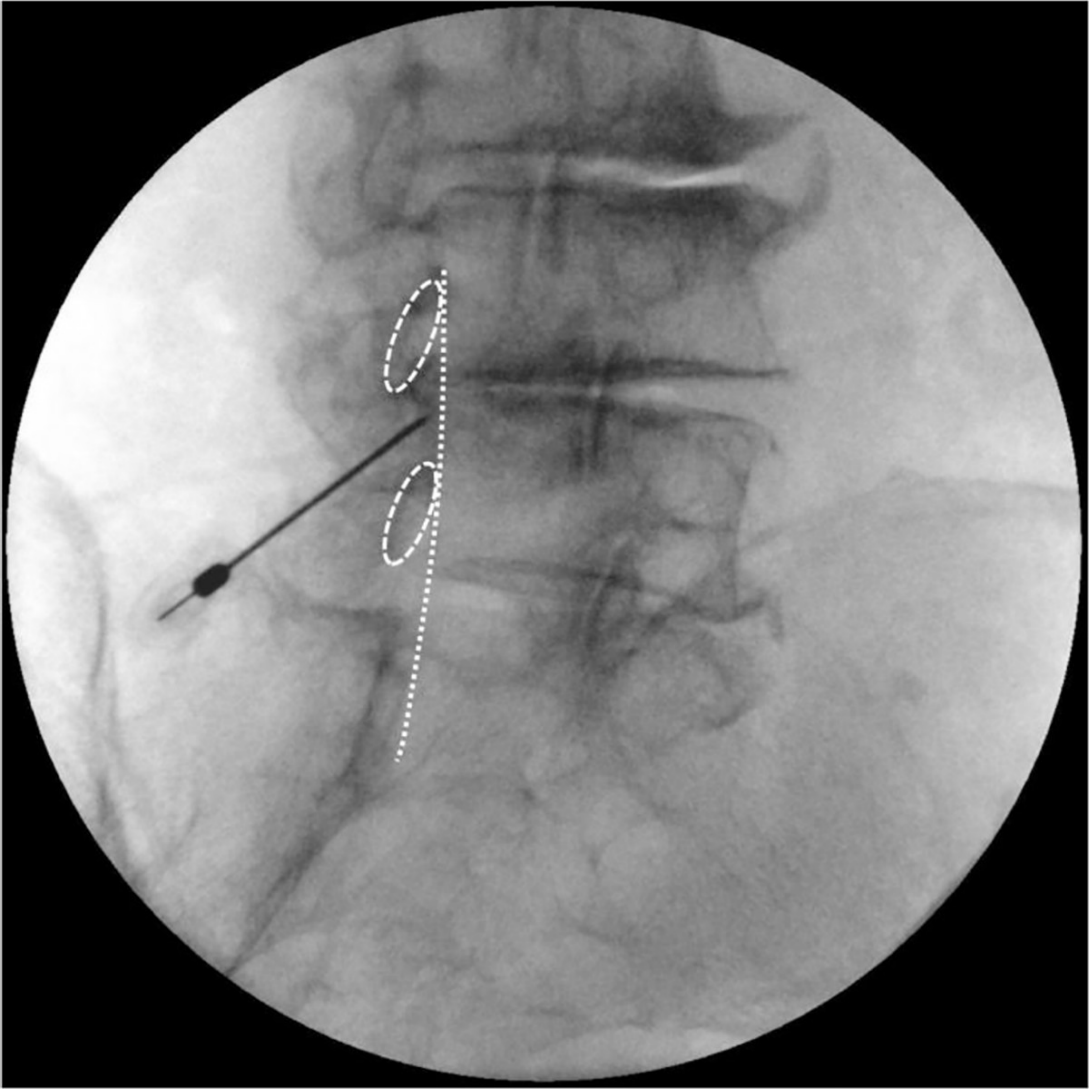
Advancement of the needle near the ventral interlaminar line (VILL). Dotted oval shapes indicate the vertebral laminas and the dotted line indicates the VILL.

The lamina and interlaminar opening in CLO view were assessed, and the needle was inserted near the VILL (Fig 4).

**Fig 5:**
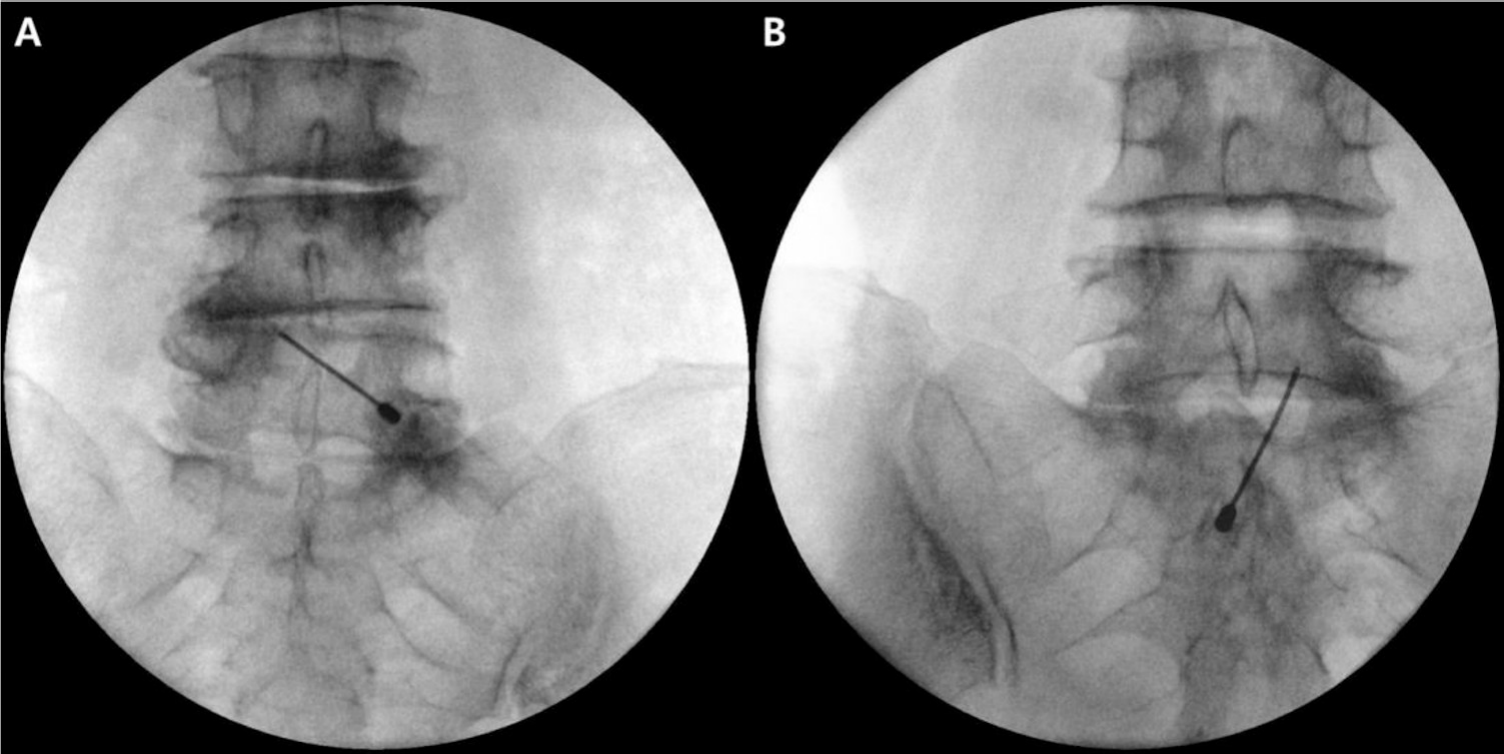
After positioning the needle near the ventral interlaminar line (VILL) on contralateral oblique (CLO) view, the needle was verified to be oriented to the pedicle of the target side in fluoroscopic anteroposterior (AP) view. Fig 5A and 5B are the same patient cases as in Fig 2A and 2B, respectively.

At this point, fluoroscopic AP view was re-examined to confirm the needle direction toward the pedicle of the target side (Fig 5). CLO view was then recreated, and the needle was advanced through the VILL.

**Fig 6:**
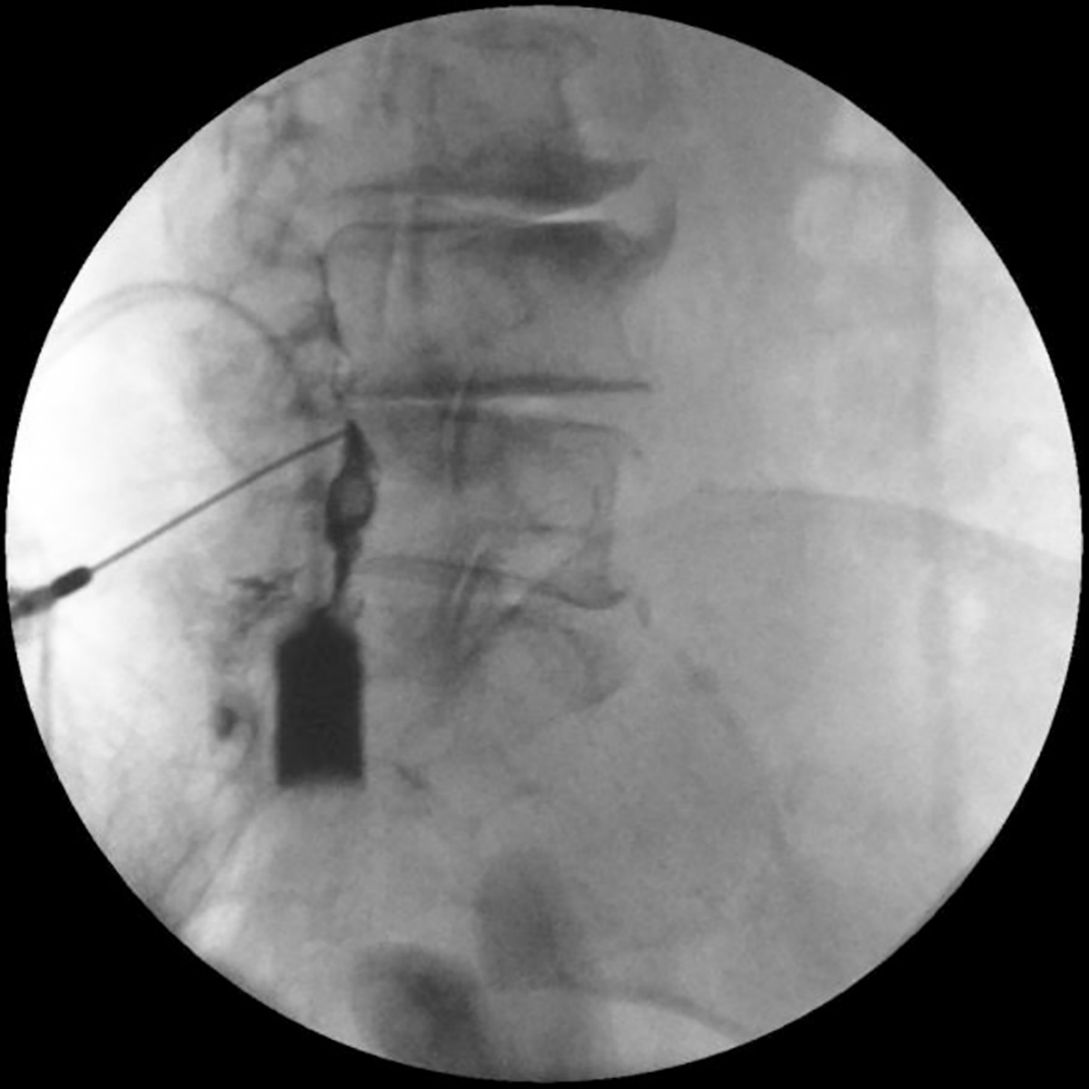
When the needle passes the ventral interlaminar line (VILL), the loss of resistance (LOR) technique is initiated, and the epidural space is confirmed with contrast medium. A syringe filled with contrast medium is shown on the screen.

Using the loss of resistance (LOR) technique, penetration of the needle into the epidural space was confirmed (Fig 6).

**Fig 7:**
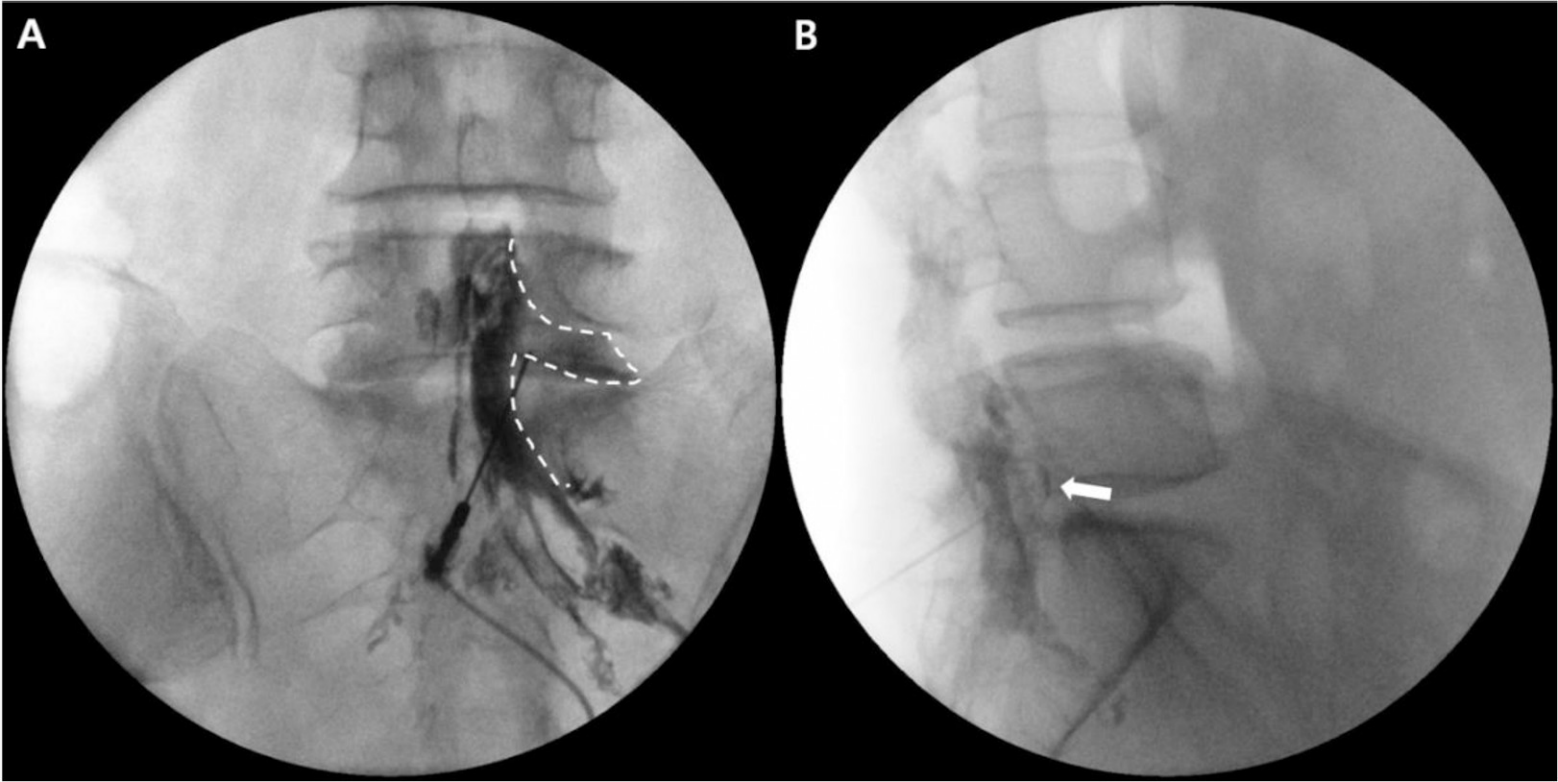
Contrast spread pattern in fluoroscopic anteroposterior (AP) view (A) and lateral view (B). The dotted line indicates contrast spread in fluoroscopic AP view. Note that the needle tip is located in the axillary portion of the right L5 nerve root. The white arrow indicates ventral spread of the contrast medium in fluoroscopic lateral view.

After confirming that the needle has entered the epidural space, the needle tip was advanced carefully to the midpoint of the spinal canal in fluoroscopic lateral view. Then, 2–3 mL of contrast medium was injected, and the contrast spreading pattern was observed (Fig 7A and 7B).

### 2.3. Outcome measurement

Age, sex, procedure direction, and lumbar spine magnetic resonance imaging (MRI) findings of the patients were collected from medical records.

The dominant pathologies of the patients were classified as bulging disc, herniated nucleus pulposus (HNP), and stenosis, as assessed by MRI findings interpreted by a radiologist. In addition, if there was a previous history of spinal surgery and pain persists, the dominant pathology was classified as failed back surgery syndrome (FBSS).

Contrast medium flow patterns were evaluated to determine accessibility to the ventral epidural space.

## 3. Results

We identified 29 patients who underwent MIL approach using CLO view. Demographic data and dominant pathologies of participants are summarized in Table 1. In all participants, the contrast medium spread into the medial epidural space. The overall rate of accessing the ventral epidural space was 93.1% (27 out of 29) (Table 2).

**Table 1:** Demographic data and dominant pathologies of participants.

Number of patients	29
Sex, n (male/female)	15/14
Direction, n (Right/Left)	17/12
Age range, years (mean±SD)	32–85 (60.85±16.19)
Dominant pathologies (n)	
Bulging disc	9
HNP	12
Stenosis	6
FBSS	2

HNP: Herniated nucleus pulposus; FBSS: Failed back surgery syndrome; SD: Standard deviation.

**Table 2:** Procedure level and accessibility to the ventral epidural space.

Procedure level (n)		Medial epidural spread rate	Ventral epidural spread success rate
L3-4	2	100% (2/2)	100% (2/2)
L4-5	10	100% (10/10)	90.0% (9/10)
L5-S1	17	100% (17/17)	94.1% (16/17)
Total	29	100% (29/29)	93.1% (27/29)

Procedure-related complications such as epidural hematoma, infection and dural injury were not observed.

## 4. Discussion

In the presence of degenerative bony changes, there might be difficult for the needle to pass through the intervertebral foramen with the TF approach and thus the injectate may not be able to deliver to the target epidural space. In these cases, with the MIL approach, the needle can be advanced through a relatively wide interlaminar opening, allowing the injectate to easily access the target epidural space [5].

However, during the conventional IL approach or the MIL approach, there are times when the needle position and landmarks, such as the lamina and interlaminar opening, are difficult to identify in the fluoroscopic lateral view [7], making it difficult to determine further advancement of the needle. Furthermore, in cases of severe degeneration or scoliosis, it may be more difficult to obtain the clear images in the fluoroscopic lateral view.

In contrast, CLO view requires less precise alignment, and the lamina and interlaminar opening can be distinguished much more clearly compared to fluoroscopic lateral view [6]. The superiority of CLO view over fluoroscopic lateral view when identifying the needle tip has been reported [8].

Previous CLO view-related studies aimed at placing the needle tip in the midline of the interlaminar opening in fluoroscopic AP view [6–8]. The proper angle for creating CLO view at the lumbar level has been reported to be 45 degrees [8].

Since the final target location of the needle tip is not at the midline of the epidural space in the MIL approach, the VILL in CLO view may not coincide with the precise beginning of the epidural space during the MIL approach. However, CLO view can be used to obtain clearer fluoroscopic images that determine when the LOR technique is initiated. With the needle in the vicinity of the VILL, the operator can make the decision to initiate the LOR technique.

This process can reduce the possibility of false signs of the epidural space and can reduce uncertainty about further needle advancement. Therefore, we believe that CLO view can be useful even when the lateral side of the spinal canal is the target location of the needle tip, as in the MIL approach.

To decrease the risk of dural injury, the original MIL approach technique recommends that needle be advanced toward the pedicle and touch the superomedial border of the interlaminar opening in fluoroscopic anteroposterior (AP) view before entering the epidural space [5].

However, the CLO view allows for clear identification of the lamina and interlaminar opening, allowing the operator to advance the needle into the epidural space without touching bone. If the appropriate laterality of the needle direction is obtained in fluoroscopic AP view, the needle can enter the axillary portion of the exiting nerve root.

In the present study, the ventral accessibility (93.1%) was slightly higher than in the previous MIL study (84.6%) [5]. This might be due to the improved accuracy of needle access by using CLO view.

This study had several limitations. First, the sample size of this study was very small. Second, this study was a retrospective analysis without a control group. In addition, since this study has focused on the technical aspects of the procedure to improve the safety and reliability of the MIL approach, further researches on the clinical outcome would be needed.

The MIL approach may be technically difficult at levels above L3-4 because the interlaminar openings are relatively small. However, lumbar pathologies mainly occur at lower lumbar levels such as L4-5 and L5-S1 [9], and since these levels have relatively wide interlaminar opening, the MIL approach can be implemented easily.

## 5. Conclusions

In conclusion, using CLO view, uncertainty can be reduced during the MIL approach to shorten procedure time and improves safety. Further studies are needed to demonstrate this advantage.

## Supporting information

S1 File (XLSX)
